# Low uptake of silica nanoparticles in Caco-2 intestinal epithelial barriers

**DOI:** 10.3762/bjnano.8.141

**Published:** 2017-07-07

**Authors:** Dong Ye, Mattia Bramini, Delyan R Hristov, Sha Wan, Anna Salvati, Christoffer Åberg, Kenneth A Dawson

**Affiliations:** 1 Centre for BioNano Interactions, School of Chemistry and Chemical Biology, University College Dublin, Belfield, Dublin 4, Ireland; 2 present address: AbbVie Deutschland GmbH & Co KG, Brain Delivery at Neuroscience Discovery, Knollstraße, 67061 Ludwigshafen, Germany; 3 present address: Center for Synaptic Neuroscience and Technology, Istituto Italiano di Tecnologia, L.go Rosanna Benzi 10, 16132, Genova, Italy; 4 present address: Groningen Research Institute of Pharmacy, University of Groningen, Antonius Deusinglaan 1, 9713 AV Groningen, The Netherlands

**Keywords:** Caco-2, differentiation and polarisation, epithelial cell barrier, microscopy imaging, particle interaction, uptake and localisation

## Abstract

Cellular barriers, such as the skin, the lung epithelium or the intestinal epithelium, constitute one of the first obstacles facing nanomedicines or other nanoparticles entering organisms. It is thus important to assess the capacity of nanoparticles to enter and transport across such barriers. In this work, Caco-2 intestinal epithelial cells were used as a well-established model for the intestinal barrier, and the uptake, trafficking and translocation of model silica nanoparticles of different sizes were investigated using a combination of imaging, flow cytometry and transport studies. Compared to typical observations in standard cell lines commonly used for in vitro studies, silica nanoparticle uptake into well-developed Caco-2 cellular barriers was found to be very low. Instead, nanoparticle association to the apical outer membrane was substantial and these particles could easily be misinterpreted as internalised in the absence of imaging. Passage of nanoparticles through the barrier was very limited, suggesting that the low amount of internalised nanoparticles was due to reduced uptake into cells, rather than a considerable transport through them.

## Introduction

An overall conclusion from a multitude of nanoparticle-cell in vitro studies is that nanoparticle uptake into cells is readily achieved in most cell types, simply by exposing cells to media containing nanoparticles. Preventing nanoparticles from entering cells seems more challenging, though in the lab this may be achieved by depleting cellular energy, thereby blocking active cellular pathways [1–3]. The unique capacity of nanoparticles to enter cells, although promising for nanomedicine [4–9], has caused concerns of nanosafety in relation to unintended exposure to nanoparticles used in different technological applications (food additives, paintings, and others) [10–13].

Along different exposure routes, cellular barriers, such as the skin, the lung epithelium, the intestinal epithelium or the endothelium (including the blood-brain barrier), constitute one of the first sites of interactions of nanoparticles, whether intended as nanomedicines or not, with organisms. Thus in addressing the potential use of nanoparticles for nanomedicine or the potential hazards of nanomaterials, it is fundamental to consider how nanoparticles interact, are processed by and transported across such barriers [14–16].

Specialised cellular layers also represent a more complex system compared to single isolated cells commonly used for in vitro studies. Indeed, cellular layers can develop tight junctions and become polarised. Polarised cell barriers are known to activate cellular processes and pathways which are not fully developed, or even present, in isolated cells [17–18]. Thus they allow investigation of nanoparticle-cell interactions in a situation closer to that in vivo.

Among several well established examples of in vitro cellular layers, the human colon carcinoma epithelial cell line Caco-2 constitutes a reliable model of the intestinal barrier, which has been widely used to predict function and bioavailability of compounds in drug delivery. In vitro drug transport studies with differentiated Caco-2 barriers are usually performed using transwell systems that allow distinguishing the apical and basal side of the developed monolayer, after polarisation and tight junction expression have been achieved [19–21].

Caco-2 cells have been used in the literature to investigate the potential toxic effects of a range of nanoparticles, including microporous silicon [22], silica [23–28] and zinc oxide [25]. Though such studies have mainly been performed on undifferentiated cells, rather than Caco-2 barriers, interestingly, there are suggestions that the response is different in overnight cultures and cells grown for 10 days [25]. Uptake into Caco-2 cells has been reported for silica [23–24,26,28], polystyrene [29], chitosan [30], poly(lactic-*co*-glycolic acid) [31–33] and poly(lactic-*co*-glycolic acid) and poly(lactic acid) with attached poly(ethylene glycol) [31–32] nanoparticles and to be temperature-dependent [29–33]. Despite uptake, transport across differentiated Caco-2 barriers (grown for 21 days) has been shown to be very limited for nanoparticles such as microporous silicon [22], silica [23], poly(lactic-*co*-glycolic acid) [33] and carboxylated and aminated polystyrene [34].

We hypothesize that the low translocation observed in Caco-2 barriers results from a low uptake into the cells, an uptake that depends on cellular differentiation and polarisation. Additionally, though it is known that biomolecules adsorbed to nanoparticles affect their cell adherence [35], uptake [26,35] and translocation [34], we wish to clarify their role in the full process, from adherence to translocation, in both undifferentiated and mature Caco-2 barriers.

To this end, model silica nanoparticles of different sizes, for which information on uptake and intracellular distribution in typical in vitro cell lines is already available [36–37], were exposed to differentiated Caco-2 barriers. In order to determine the role of molecules adsorbed on the nanoparticles from the surrounding environment, nanoparticle exposure was performed in the absence and presence of foetal bovine serum as a model biofluid. Indeed, it is crucial to utilize some type of biofluid, because it has been shown that in its absence the high surface energy of the bare nanoparticle surface may cause cell damage [36,38]. This is unlikely to happen in any realistic nanoparticle exposure scenario, where biomolecules adsorb to the nanoparticle to form a nanoparticle biomolecular corona which effectively protects the cells from such immediate damage [39–41]. Though the intestinal lumen is not in direct contact with blood serum, since the cells are normally cultured in vitro in the presence of foetal bovine serum, we used foetal bovine serum as a starting point of our work. Uptake into and transport across the cellular barriers were investigated with a combination of flow cytometry and fluorescence and electron microscopy imaging. Transport studies across transwell systems, typically used for the study of drug transport across similar layers, were also applied. The combination of these different methods was essential to avoid easy misinterpretations of the outcomes.

## Results and Discussion

### Particle physicochemical characterisation

Green fluorescent silica nanoparticles (SiO_2_-NPs) of 50 and 150 nm diameter were synthesized according to previous literature [42]. In order to remove eventual free fluorescent dye releasing from the labelled nanoparticles [3,43–44], the nanoparticle stocks were cleaned (by pelleting and resuspending in fresh buffer) prior to experiments with cells. Sodium dodecyl sulphate polyacrylamide gel electrophoresis (SDS-PAGE) confirmed that for both nanoparticles, no labile dye was present in the nanoparticle dispersions (Supporting Information File 1, Figure S1). Based upon previous experience, we limited exposure times to 6 hours in order to reduce the risk of released free dye and fragmentation of the nanoparticles, stemming from partial solubility in cell culture medium, which could confuse uptake and transport studies [42].

Nanoparticle size and state of agglomeration in the different media of interest were determined by differential centrifugal sedimentation (DCS). The characterisation of one batch is summarised in Table 1 (Supporting Information File 1, Figures S2 and S3 show corresponding distributions). DCS of both nanoparticles suspended in water shows that the sizes approximately matched the intended sizes, though the 150 nm nanoparticles exhibited two populations (of similar size). In Hank's balanced salt solution (HBSS), both in the absence and presence of foetal bovine serum (FBS), DCS shows that the samples remain dispersed, with no signs of agglomeration, though naturally serum protein adsorption increases the sizes. Table 1 also shows auxiliary dispersion characterisation by dynamic light scattering (DLS), leading to the same conclusions.

**Table 1:** Physicochemical characterisation of the 50 nm and 150 nm SiO_2_-NPs in different media. Nanoparticle dispersion (100 µg/mL) in water, serum free HBSS and HBSS supplemented with 10% foetal bovine serum (FBS) were characterised by differential centrifugal sedimentation (DCS) and dynamic light scattering (DLS) as described in the Experimental section. The table shows the apparent diameter and peak full width at half maximum (FWHM) obtained by DCS, together with the results obtained by cumulant analysis of the DLS data (*z*-average diameter and polydispersity index, PDI). The corresponding distributions are shown in Supporting Information File 1, Figures S2 and S3. For DLS results, errors represent standard deviation of 4 replicates (11 runs each).

Nanoparticle	Medium	DCS apparent diameter (nm)	DCS FWHM (nm)	Diameter (*z*-average, nm)	PDI

50 nm SiO_2_	water	41	20	76^a^	0.159^a^
	HBSS	44	23	79 ± 1^b^	0.17 ± 0.02^b^
	HBSS, 10% FBS	50	27	102 ± 3^c^	0.42 ± 0.07^c^
150 nm SiO_2_	water	160, 183^d^	60^d^	154 ± 4	0.03 ± 0.03
	HBSS	159, 181^d^	60^d^	190 ± 2	0.02 ± 0.01
	HBSS, 10% FBS	149, 170^d^	60^d^	209 ± 2	0.18 ± 0.02

^a^Data (for equivalent batch) reproduced from [42]. ^b^The presence of larger particles in the distribution of sizes (see DCS data in Supporting Information File 1, Figure S3) will bias the DLS average towards larger sizes due to the stronger scattering from larger particles (the scattering intensity grows strongly with particle size). ^c^A second small peak around 10 nm due to the presence of proteins was also visible (Supporting Information File 1, Figure S2) which explains the high average diameter and PDI. This was not visible for the larger particles, likely due to the substantially stronger scattering stemming from them. ^d^Multiple peaks were observed by DCS (Supporting Information File 1, Figure S3) and both peak positions are reported together with the combined width.

Nanoparticle dispersions were prepared with and without FBS protein in order to test SiO_2_-NP uptake and interactions with the Caco-2 barrier in the presence and absence of a nanoparticle corona. The corona forming on the SiO_2_-NPs at different concentrations of serum was also characterised (Supporting Information File 1, Figure S4) and results were in agreement with previous findings on similar nanoparticles [45].

### Formation and characterisation of Caco-2 barriers

Caco-2 cells were cultured on typical transwell systems for 21 days as described in the Experimental section, in order to ensure formation of a polarised cell monolayer and development of tight junctions [19,46–47]. Typical transmission electron microscopy (TEM) images of the obtained barriers are shown in Supporting Information File 1, Figure S5. Cells with the nuclei in a basal position and apical microvilli were observed, and tight junctions were well expressed between the confluent cells in the monolayer.

Transepithelial electrical resistance (TEER) was used to further confirm barrier formation and test the integrity of the barriers before and after exposure to the nanoparticles. TEER measurements confirmed that the Caco-2 cell-monolayers exerted high resistance values (up to 1000 Ω·cm^2^ on a 12-well transwell, growth area 1.12 cm^2^) after 21 days of culture, comparable to what has been reported in literature [46–48]. After exposure to 50 nm or 150 nm SiO_2_-NPs, only a small decrease in resistance was observed (Supporting Information File 1, Figure S6), suggesting no major effect of the particles on the overall barrier integrity.

### Nanoparticle uptake in Caco-2 barriers cultured for 4 and 21 days

Flow cytometry was used to quantify cell fluorescence intensity due to association of the fluorescent SiO_2_-NPs with the cells. In order to test if cell polarisation/differentiation plays a role in controlling nanoparticle association with the Caco-2 barriers, we performed this experiment after culturing the cells for 4 days or 21 days. After 4 days of culture, Caco-2 cells reach complete confluence, but their resistance is still low compared to their 21-day counterpart (Supporting Information File 1, Figure S7); 21 days of culture is needed for the full establishment of a polarised barrier with fully developed tight junction expression. We exposed the cultures to the two sizes of SiO_2_-NPs, in both cases with an excess of particles compared to the number of cells and at the same concentration (in mass per volume, implying a different concentration in number per volume).

**Figure 1:**
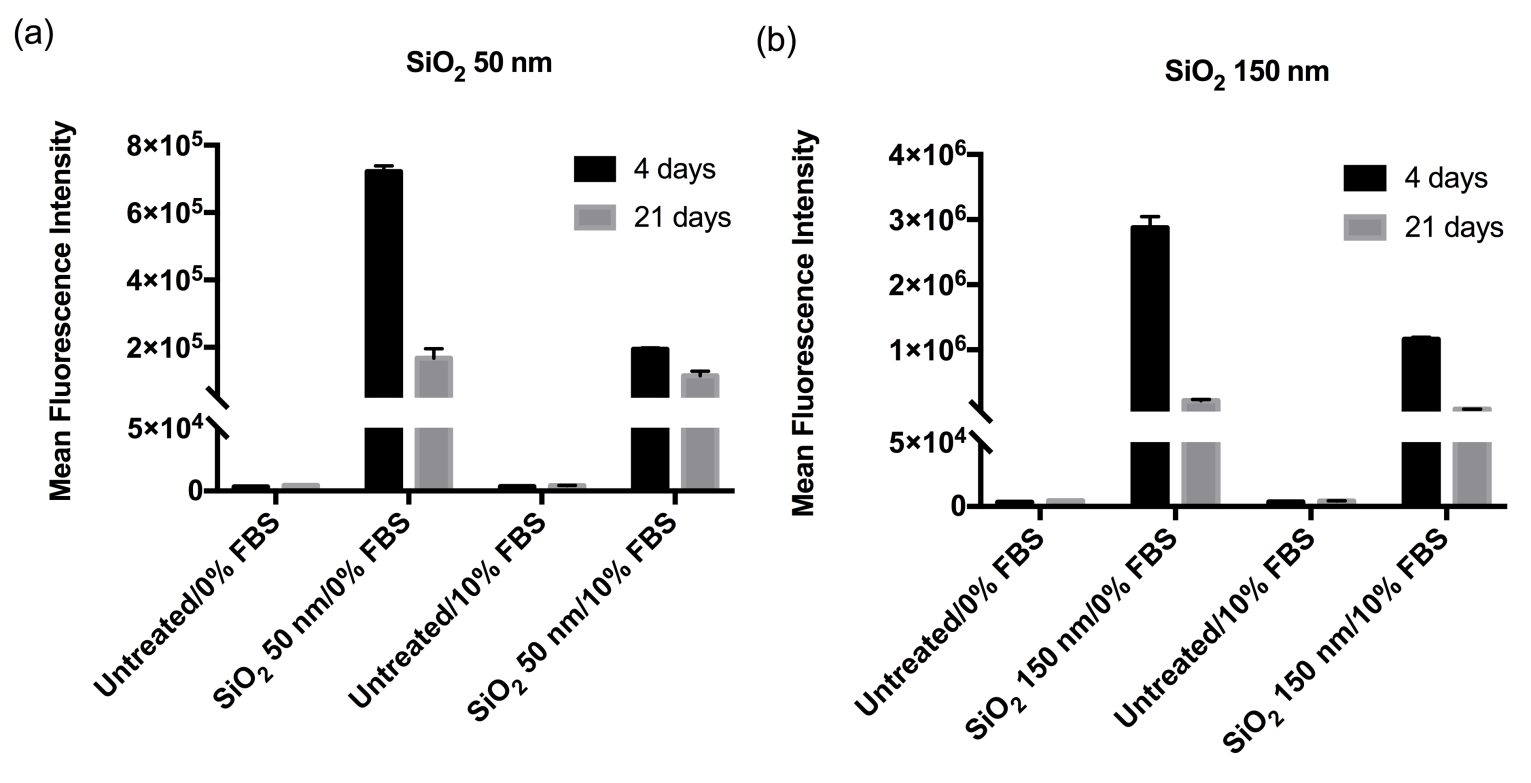
50 nm and 150 nm SiO_2_-NP association with Caco-2 barriers. Caco-2 barriers cultured for 4 and 21 days were exposed for 6 h to (a) 50 nm and (b) 150 nm SiO_2_-NPs (100 µg/mL) in the absence and presence of 10% serum, prior to cell fluorescence measurements by flow cytometry. Results are presented as the mean cell fluorescence intensity (due to nanoparticles) with error bars representing the standard error of the mean of 3 technical replicates (Supporting Information File 1, Figure S8 shows a comparison of three independent experiments). One may observe a general trend of a larger nanoparticle association to barriers cultured for 4 days compared to those cultured for 21 days, as well as a trend of larger association in the absence of serum. Note that the two nanoparticles are loaded with different amounts of fluorescent dye so no (direct) absolute comparison may be made between the two sizes.

The cell fluorescence distributions obtained after exposure to nanoparticles were rather broad (in comparison to what is observed for similar particles in single cells [3,37]) indicating rather different behaviours for cells within the same population. Figure 1 shows a clear qualitative trend where Caco-2 barriers cultured for 4 days exhibit a higher cell fluorescence intensity compared to those cultured for 21 days under the same seeding conditions. This is true regardless of the presence or absence of serum, and regardless of the two particle sizes. Part of this effect could be due to an increased packing of cells, manifesting itself as a decreased surface area per cell between 4 and 21 days, and thus a resulting lower association to the cell membrane and consequent uptake. However, it is difficult to imagine that this could amount to a difference that is sometimes as large as an order of magnitude (cf. 150 nm SiO_2_ dispersed in serum-free medium in Figure 1b). Furthermore, one may argue that the development of microvilli in the barriers cultured for 21 days increases the available surface area for contact with the nanoparticles, thus counteracting the eventual decrease in surface area due to increased packing of cells. This suggests that the observation is likely a genuine effect of cell polarisation/differentiation and that indeed nanoparticle interactions with well-developed cell layers can be very different compared to what is observed in under-established cell barriers, even when confluent.

Finally, cell association of nanoparticles dispersed in the absence of serum is stronger compared to in the presence of serum (Figure 1). This observation is consistent with literature, where the presence of serum typically confers a lower association with cells due to the formation of a biomolecular corona [41] reducing the high surface energy of the bare particle surface [36,38].

We next performed time resolved experiments (Supporting Information File 1, Figure S9) where Caco-2 barriers (cultured for 21 days) were exposed to nanoparticles for 6 h, the nanoparticle source removed and the cells cultured for a further 4 h prior to cell fluorescence measurement by flow cytometry (that is, a 6 h “pulse” followed by a 4 h “chase”). In the majority of cases, one observes (Supporting Information File 1, Figure S9) a trend where the cell fluorescence decreases during the 4 h without nanoparticles present, suggesting that the association of nanoparticles with cells is (at least partly) only transient.

To better understand the observed behaviour and investigate further particle uptake and localisation under the different conditions, we turned to fluorescence imaging. Cell barrier growth might be affected by different growth support material [49], but culturing the Caco-2 barriers on a hard surface (coverslides) instead of a porous transwell, allows correlating the imaging results to those coming from flow cytometry (where the barriers were cultured in 12-well plates).

**Figure 2:**
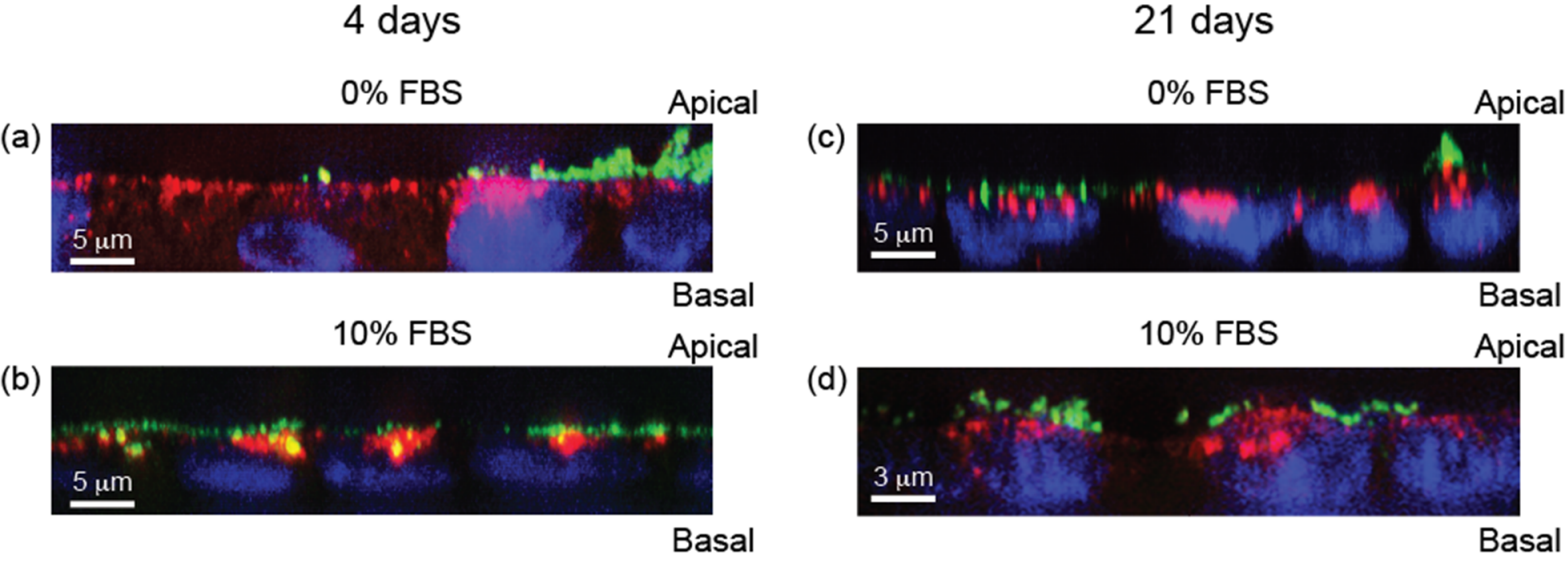
Confocal fluorescence cross-section images of Caco-2 barriers exposed to 50 nm SiO_2_-NPs. Caco-2 barriers cultured for 4 (a,b) and 21 days (c,d) were exposed for 6 h to 100 µg/mL 50 nm SiO_2_-NPs in the absence of serum (0% FBS; a and c) and presence of serum (10% FBS; b and d). The nanoparticles are shown in green. Lysosomes and nuclei were stained by Lysosomal-Associated Membrane Protein 1 (LAMP1) antibody (red) and DAPI (blue), respectively.

Figure 2 shows cross-sections taken from confocal imaging of Caco-2 cell barriers cultured for 4 and 21 days following exposure to 50 nm SiO_2_-NPs for 6 h. A first clear outcome is that, in general, not many 50 nm SiO_2_-NPs (green) could be visualized inside cells, regardless of polarisation/differentiation of the cells. Rather, most nanoparticles were localized on the apical cell membranes outside the cells. These observations allow us to reconcile the results from flow cytometry showing a substantial association of nanoparticles with cells during continuous uptake (Figure 1) together with a loss of association after exposure is stopped (Supporting Information File 1, Figure S9). There is indeed a large association of nanoparticles with cells, but this association is transient, because most nanoparticles are not internalised but adhering to the outside of the cells [25,35] and simply desorb once the nanoparticle source is removed. The interpretation is then that the signal measured by flow cytometry is largely coming from nanoparticles adhering to the outside of the cells, rather than internalised. We note that sample processing before flow cytometric assessment may remove some of the nanoparticles adhered on the outside of the cell. Thus, the flow cytometry results are actually biased towards showing a lower adsorbed amount, so the effect may actually be larger than observed. In addition, the fact that a significant number of particles still remains adsorbed to cells suggests that the adsorption is fairly strong, consistent with the slow desorption process (Supporting Information File 1, Figure S9).

The lysosomes were also stained (LAMP1 antibody; red) as previous studies have shown significant lysosomal colocalisation for several nanoparticle/cell systems [2–3,37,50]. Lysosomal staining showed that some of the (few) internalised nanoparticles were found in the lysosomes, and this was more evident for the 50 nm SiO_2_-NPs in Caco-2 barriers cultured for 4 days compared to those cultured for 21 days.

Transmission electron microscopy (TEM) imaging was used to further explore nanoparticle localisation in the Caco-2 barriers. We confirmed that 6 h exposure led to very little internalisation of particles in the Caco-2 barriers. In contrast, most of the observed nanoparticles were found outside the cells, associated with the microvilli (Supporting Information File 1, Figure S10). This observation is clearly in agreement with the high amount of fluorescence at the apical surface of the barriers seen using light microscopy (Figure 2). It should be noted that such particle association might be sensitive to the removal by washing and other steps in the sample preparation procedure for flow cytometry, which could explain the broad variability of our flow cytometry data (Supporting Information File 1, Figure S8).

**Figure 3:**
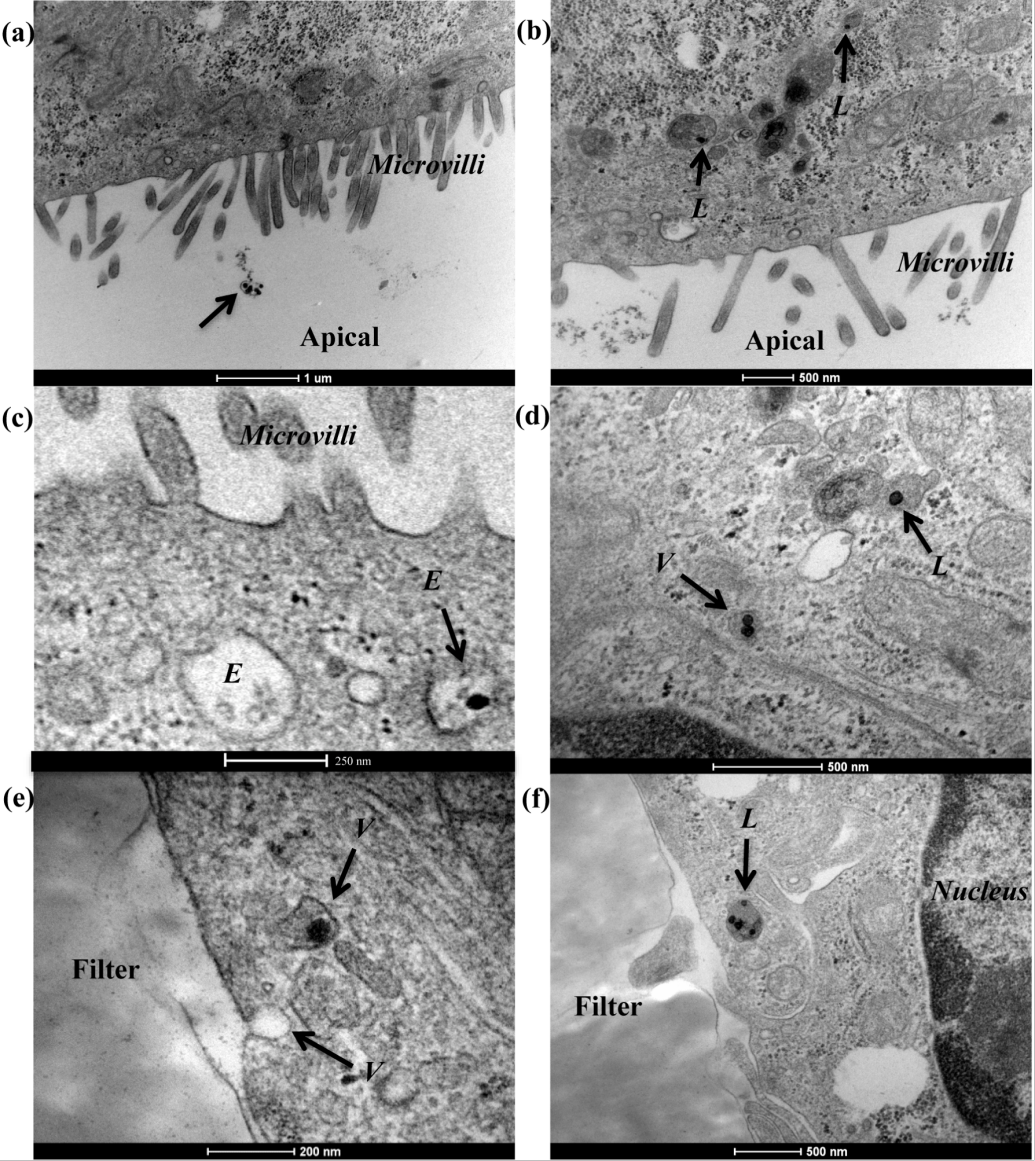
Transmission electron micrographs of Caco-2 barriers after exposure to 50 nm SiO_2_-NPs. Caco-2 barriers cultured for 21 days were exposed for 9 h to 100 µg/mL 50 nm SiO_2_-NPs dispersed in (a,b) the absence of serum, and (c–f) the presence of 10% FBS. The arrows indicate some extracellular NPs and a few NPs observed inside the cells in different vesicles along the endolysosomal pathway. Abbreviations: *E*, endosome; *L*, lysosome; *V*, vesicle.

**Figure 4:**
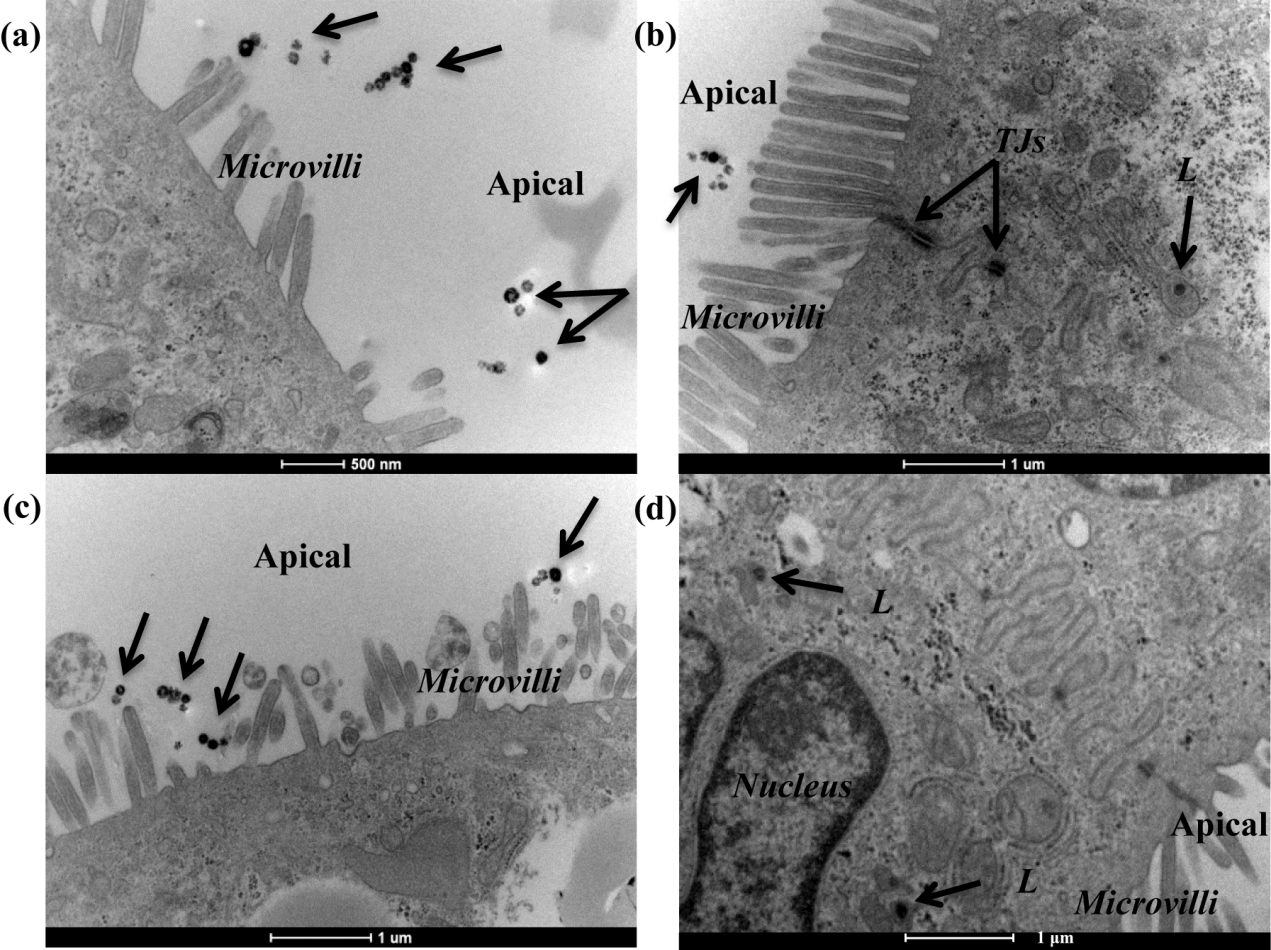
Transmission electron micrographs of Caco-2 barriers after exposure to 150 nm SiO_2_-NPs. Caco-2 barriers cultured for 21 days were exposed for 9 h to 100 µg/mL 150 nm SiO_2_-NPs dispersed in (a,b) the absence of serum, and (c,d) the presence of 10% FBS. The arrows indicate extracellular NPs and some NPs inside vesicles along the endolysosomal pathway. Abbreviations: *L*, lysosome; *TJs*, tight junctions.

Due to the low amount of SiO_2_-NPs internalised after 6 h, we prolonged the exposure time to 9 h hoping for a better chance to capture nanoparticle-related intracellular events. Some illustrative images for 50 nm and 150 nm SiO_2_-NPs are shown in Figure 3 and Figure 4, respectively. The particles are clearly visible in electron microscopy due to their high density, as also observed in our previous work [14–15,37,51]. Overall, consistent with the above results, very few nanoparticles were found inside the cells. Nevertheless, in the absence of serum, 50 nm SiO_2_-NPs can be seen in the vicinity of microvilli (Figure 3a) and a few nanoparticles were also found in lysosomes (Figure 3b). For 50 nm SiO_2_-NPs dispersed in 10% serum, nanoparticles were found enclosed in vesicles along the endo-lysosomal pathways, including in endosomes (Figure 3c) and lysosomes (Figure 3d and f). Furthermore, Figure 3e shows a single nanoparticle within a vesicle close to the basolateral membrane, where another vesicle has docked. Although rare, we occasionally made such observations, both using TEM and fluorescence microscopy (Supporting Information File 1, Figure S11). Not wishing to overstate these observations, such vesicular events may suggest rare events of transcytosis. Further studies need to be performed in order to fully address whether or not rare transcytosis events may occur.

Some internalisation events were also seen for 150 nm SiO_2_-NPs (Figure 4), with particles found in vesicles and lysosomes in both 0 and 10% serum (Figure 4b and d, respectively) as described for the smaller nanoparticles (Figure 3).

Finally, we performed transport studies to determine whether the low amount of internalised nanoparticles observed by the different microscopy methods was due to low uptake or fast passage through the barrier. We found that the amount of SiO_2_-NPs in the basolateral chamber was less than 1 wt % of the applied apical dose, regardless of serum concentration or its presence (Supporting Information File 1, Figure S12). Such a low transport ratio is likely below the detection limit of the experimental setup, and our conclusion is simply that nanoparticle transport across the barrier is negligible under all conditions investigated, at least during this limited time-scale. This conclusion is in agreement with other similar studies [22].

## Conclusion

In the work presented here, we studied the interaction of model silica nanoparticles with Caco-2 barriers. Nanoparticle association with the Caco-2 barriers was considerably influenced by the state of polarisation/differentiation of the cells, with a consistently lower association of nanoparticles with polarised/differentiated barriers (that is, with barriers cultured for 21 rather than 4 days). We argue that this effect cannot be explained solely by a reduction in exposed cell surface area when a tight barrier is formed, and that the formation of microvilli would anyway counteract such an effect by providing a larger surface area for nanoparticle association. Thus the observation seems to be a genuine biological response due to barrier polarisation/differentiation. Full development of an extracellular matrix with mucus on the apical cell side could also constitute a further obstacle for particle association with the barrier.

Furthermore, we found that the majority of nanoparticles associated with the cells were adsorbed to the outer cell membranes, rather than being internalised by the Caco-2 barriers. Thus, results from flow cytometry exhibit a substantial signal which, in isolation, could be misinterpreted for nanoparticle accumulation, but which fluorescence and electron microscopy imaging show is (predominantly) due to nanoparticle adsorption to the outer cell membrane. Nevertheless, imaging shows some nanoparticle internalisation into the Caco-2 barriers, commonly leading to lysosomal accumulation. Transport through the barrier was found to be very low, suggesting that the low amount of internalised nanoparticles was due to a low internalisation efficiency in the barriers rather than to a substantial transport across it.

The low degree of internalisation is particularly striking in comparison to our previous observations of a substantial uptake of silica nanoparticles into (single) lung cells [37]. Moreover, we have previously found sizable uptake of silica and polystyrene nanoparticles into another type of barrier, namely an in vitro model of the human endothelial blood brain barrier [14–15,51–52]. Thus, the low degree of uptake observed in the Caco-2 barrier may be a characteristic of this type of barrier and could be related to the more complex polarised nature of thicker epithelial layers. Alternatively, it could be connected to the presence of a rich extracellular matrix, which may increase nanoparticle association to the apical side of the barrier, but also impede further interactions with the actual cell membrane, thereby lowering nanoparticle uptake.

We note that our results are qualitative and that there remain many interesting strands for future research. Thus the substantial nanoparticle absorption to the outside of the cells is a concern from a purely technical standpoint, potentially making quantitative results highly dependent upon the nature of washing and other sample preparation steps (indeed, we found quantitative reproducibility to be an issue, as already noted). Quantitative and detailed elucidation of the adsorption will have to be based on more advanced methodology. Similarly, the observation of a highly heterogeneous cell population is interesting from a scientific point of view, but also complicates the interpretation of imaging snapshots which do not sample all the variation of the population. Again, a different methodology will be needed to understand and capture this heterogeneity in detail.

There could be broader implications of the low degree of internalisation into the Caco-2 barrier. In the context of nanomedicine, the low internalisation could suggest that oral administration routes may lead to poor transport across the intestinal epithelium. For nanosafety, toxic responses measured for single cell cultures (where internalisation is usually substantial) may have exaggerated effects which will not be present for real barriers, where a lower dose is accumulated. On the other hand, lower internalised doses have also been found to activate different cellular pathways compared to those observed at higher nanoparticle loads [53]. Thus the use of cellular barriers in future could allow obtaining a more complete picture of the possible outcomes of nanoparticle-cell interactions. Nevertheless, even when low internalisation is observed, it will be important to consider final fate of the internalised load, if export or degradation are present, and potential effects of bioaccumulation over chronic exposure.

## Experimental

### Nanoparticle characterisation

Green fluorescently labelled SiO_2_-NPs of 50 and 150 nm diameters were synthesized in house according to previous literature [42]. Particle dispersions were characterised at 100 µg/mL in 0% and 10% foetal bovine serum (FBS) in Hank's balanced salt solution (HBSS) buffer (with 25 mM 4-(2-hydroxyethyl)-1-piperazineethanesulfonic acid (HEPES), pH 7.4) at 25 °C, using a Malvern Zetasizer Nano ZS90 (Worcestershire, UK) in order to measure the hydrodynamic diameter by dynamic light scattering (DLS). The 150 nm particles were also measured in water. Further characterisation was performed using differential centrifugal sedimentation (DCS) on a CPS Disk Centrifuge DC 24,000. A disc speed of 18,500 revolutions per minute (RPM) was used and an 8–24% water or HBSS buffer sucrose gradient was injected (settings optimized for size range analysis 0.03–1 µm). A 476 nm polyvinyl chloride (PVC; Analytik UK) commercial standard was used to calibrate the instrument before each measurement. Each gradient was checked by running the PVC standard as a sample and comparing to a database control. 100 µL of standard was injected before each measurement to calibrate the instrument, followed by 100 µL of the undiluted particle dispersion.

### Cell culture and exposure to nanoparticles

Caco-2 epithelial cells (supplied by European Collection of Authenticated Cell Cultures) were cultured in complete Dulbecco's Modified Eagle Medium (cDMEM) supplemented with 10% FBS, 5 mL MEM non-essential amino acids (100 X), 5 mL Penicillin-Streptomycin (100 X, 10,000 units Penicillin and 10,000 µg Streptomycin) in a cell incubator at 37 °C in a humidified 5% CO_2_ atmosphere. Cells were trypsinised from the flasks when 80–90% confluence was reached. For transwell transport studies, 2 × 10^5^ cells in 0.5 mL cDMEM were seeded onto the apical chamber of a 3.0 μm poly(tetrafluoroethylene) (PTFE) membrane insert (1.12 cm^2^ growth area), with 1.5 mL cDMEM in the basolateral chamber. After 5–16 h in the cell incubator, the apical medium was replaced by fresh cDMEM in order to remove non-adherent cells and avoid formation of multiple layers. Then the cells were grown a further 4 or 21 days, changing the medium in both chambers every second day (in the first 15 days), or every day (from 15 to 21 days). A layer of confluent cells was obtained after 4 days of culture; polarisation and tight junction expression were fully developed after 21 days. For flow cytometry experiments, cells were seeded in 12-well plates (approximately 3.9 cm^2^ growth area), and the cell density adjusted for the larger growth area in order to achieve similar growth conditions as for the transwell systems. Caco-2 cells were then grown and maintained as described above.

Prior to exposure to cells, as an additional precaution, the nanoparticle stocks were cleaned against the eventual presence of free labile dye by centrifugation at 20,000 relative centrifugal force (RCF) for 30 min, followed by resuspension in fresh buffer and sonication for 3 min in a bath sonicator [42]. Caco-2 barriers were equilibrated in HBSS buffer at 37 °C for 30–60 min, and then exposed to the nanoparticle dispersions both including and excluding 10% serum. Nanoparticle dispersions were freshly prepared by dilution to the final concentration for cell exposure (100 µg/mL) in HBSS buffer in the presence and absence of 10% FBS. Exposure to cells was performed by replacement of the HBSS with the nanoparticle dispersions.

### Flow cytometry

Uptake studies by flow cytometry were carried out on Caco-2 barriers grown for 4 and 21 days on 12-well plates as described above. The 4 and 21 days old cultures were prepared so that they were exposed on the same day to the same nanoparticle dispersions. This limits variability due to nanoparticle dispersion and all steps of sample preparation for flow cytometry. After exposure to 100 µg/mL nanoparticles dispersed in the absence and presence of 10 % FBS, the dispersions were removed from each well and cells were rinsed twice with fresh phosphate buffered saline (PBS). Then, 1 mL 0.1% trypsin-ethylenediaminetetraacetic acid (EDTA) solution was added and the cells incubated at 37 °C, 5% CO_2_ humidified atmosphere for 5–10 min to detach the cells. In some cases, where the barrier cells were difficult to remove from the plates, a second trypsinization was performed until the remaining cells were completely detached, as determined by observation under a light microscope. The detached cells were then collected from each well and the same volume of complete medium added to inhibit the trypsin. Cell pellets were harvested by centrifugation at 1,500 RPM for 3 min and resuspended in fresh PBS. In order to fix the cells, 4% formalin solution (Sigma-Aldrich) in PBS was applied for 20 min and then replaced with PBS. Prior to measurements by flow cytometry, cell samples were stabilised at 4 °C for 1–2 h in darkness. Cell fluorescence intensity was then acquired by flow cytometry using an Accuri C6 flow cytometer (15,000 cells for each sample, after exclusion of cell debris according to their forward and side scattering). The average values of the mean of the cell fluorescence distributions obtained in this way (three replicates) were calculated, together with the standard error of the mean (SEM), in order to determine nanoparticle uptake or association with cells.

### Fluorescence imaging

Glass coverslips were sterilised in 70% ethanol and placed into a 12-well plate. Caco-2 cells were seeded and grown for 4 and 21 days, as described above. After exposure to nanoparticles, the nanoparticle dispersion was removed and cells rinsed with PBS three times. Caco-2 cells were fixed and permeabilised with methanol for 4 min at −20 °C and then carefully washed with PBS three times. Antibody unspecific binding was prevented by treating the cells with a blocking buffer of 1% bovine serum albumin (BSA)-Tween PBS for 30 min (Tween 0.05%, v/v). To stain the lysosomes, cells were incubated with LAMP-1 primary antibody (Abcam; dilution 1:100) for 1 h at room temperature and then washed three times with PBS, prior to incubation with Alexa 647 secondary antibody (Invitrogen; dilution 1:300) under the same conditions. Cell nuclei were stained by incubation with 4’,6-diamidino-2-phenylindole (DAPI) for 5 min. The slides were then sealed by addition of Mowiol mounting medium (Calbiochem) and stored at 4 °C overnight. For image acquisition, tricolour visualization of cell organelles, nanoparticles and nuclei, was performed on a confocal microscope equipped with a CSU-X1 spinning disk unit (Yokogawa Electric corporation), an iXon electron-multiplying charge-coupled device (EMCCD) camera (Andor, Belfast, UK) and an inverted E-clipse microscope (Nikon, Tokyo, Japan). SiO_2_-NPs were excited with a 488 nm laser line (emission signal collected using a band pass 512 nm filter), LAMP-1 with a 561 nm laser (emission light collected with a long pass 624 nm filter) and DAPI with a 405 nm laser (emission light collected with a 448 nm filter). 40 X and 100 X Olympus UPlanSAPO oil immersion objective lenses were used. Images were acquired using Andor iQ2 software and processed using Imaris imaging software (BitPlane, Zurich, Switzerland).

### Transmission electron microscopy

After exposure to the nanoparticles as described above, Caco-2 cell monolayers grown on 3.0 μm PTFE transwell membranes were fixed with glutaraldehyde (2.5%, v/v) at room temperature for 1 h in Sorensen’s phosphate buffer, and post fixed in osmium tetroxide (1%, w/v) in de-ionised water for 1 h. The samples were rinsed in Sorensen’s phosphate buffer and dehydrated by incubation in 30%, 50%, 70%, 90% and 100% ethanol solutions. The cells were immersed in ethanol/Epon (1:1, v/v) mixture for 1 h before being transferred to pure Epon and embedded at 37 °C for 2 h. The final polymerization was carried out at 65 °C for 24 h. With a reported approach [16], ultrathin sections of 80 nm, obtained with a diamond knife using an ultramicrotome Leica U6, were mounted on copper grids, and stained with 2% uranyl acetate and 0.4% lead citrate. The sections were further examined at 120 kV with an FEI TECNAI transmission electron microscope.

## Supporting Information

File 1 Supplementary methods and figures.
