# Supplementary material for: Low uptake of silica nanoparticles in Caco-2 intestinal epithelial barriers
Source: Beilstein J Nanotechnol. 2017 Jul 7;8:1396–406. doi: 10.3762/bjnano.8.141 (PMC5530606; doi:10.3762/bjnano.8.141)
Supplement: File 1 — Supplementary methods and figures. [file Beilstein_J_Nanotechnol-08-1396-s001.pdf]

Supporting Information

for

Low uptake of silica nanoparticles in Caco-2 intestinal epithelial barriers

Dong Ye[1,2], Mattia Bramini[1,3], Delyan R. Hristov[1], Sha Wan[1], Anna Salvati[1,4], Christoffer Åberg[1,4] and Kenneth A. Dawson*[1]

Address: [1]Centre for BioNano Interactions, School of Chemistry and Chemical Biology, University College Dublin, Belfield, Dublin 4, Ireland, [2]Present address: AbbVie Deutschland GmbH & Co KG, Brain Delivery at Neuroscience Discovery, Knollstraße, 67061 Ludwigshafen, Germany, [3]Present address: Center for Synaptic Neuroscience and Technology, Istituto Italiano di Tecnologia, L.go Rosanna Benzi 10, 16132, Genova, Italy and [4]Present address: Groningen Research Institute of Pharmacy, University of Groningen, Antonius Deusinglaan 1, 9713 AV Groningen, The Netherlands

Email: Kenneth A. Dawson - kenneth.a.dawson@cbni.ucd.ie

* Corresponding author

Supplementary methods and figures

Methods

Protein corona

To isolate and characterise hard corona proteins, solutions of 100 μg/ml 50 and 150 nm SiO_2-NPs in 0%, 10%, 55% and 80% FBS in HBSS were prepared. After 1 h and 6 h incubation at 37 ^oC, SiO_2-NPs were pelleted by centrifugation at 18,000 RCF 15 ^oC for 40 min and the excess proteins in the supernatant discarded. In order to isolate only the hard corona proteins, the pellets were washed with fresh PBS and centrifuged under the same conditions for 25 min three times more. Final products were suspended in 30 μl fresh PBS with the addition of 15 μl SDS-DTT loading buffer (10:1). Around 10 μl of the final mixtures were loaded into SDS-PAGE and run at 130 V for 1 h. The gel was then stained using silver staining kits according to manufacturer instructions. Images were scanned on a Quantity One scanner. The densities of selected protein bands were analyzed with ImageJ software in order to compare their abundance under the different conditions.

Free dye electrophoresis

To screen nanoparticles for the eventual presence of a fraction of free labile dye, the silica nanoparticles used for this study (50 and 150 nm; non-modified surface) together with commercial nanoparticles of similar sizes (50 and 100 nm Kisker silica nanoparticles; non-modified surface) were subjected to SDS-PAGE electrophoresis, following methods previously described [1-3]. All the different SiO_2-NP batches were tested at 25 μg/ml in de-ionised water at room temperature, and the samples loaded on the gel by mixing the particle solution with 1/3 volume of SDS-DTT loading buffer

(10:1). Samples were run at 130 V for 1 h and a gel fluorescence image was obtained using a Typhoon scanner.

Transepithelial electrical resistance

Transepithelial electrical resistance (TEER) measurements were performed in order to determine the integrity of the Caco-2 barriers. As described in the Experimental section of the main text, Caco-2 barriers were equilibrated in HBSS buffer for 30–60 min, and then exposed to the nanoparticles dispersed in HBSS supplemented with 0%, 10%, 55% and 80% FBS at 37 °C for 6 h. Then the cells were carefully washed with pre-warmed HBSS three times, and the TEER immediately measured using an Endome chamber (World-Precision Instruments Inc., New Haven, CT, USA). Barriers treated with 0%, 10%, 55% and 80% FBS HBSS solutions only (without nanoparticles) were also characterised after 6 h exposure in order to discriminate the effects of the serum from that of the nanoparticles. Untreated controls in cDMEM were also measured. The resistance of blank transwells was subtracted from all values. The results are presented as the change of the TEER value with respect to that of untreated cells in cDMEM (TEER of treated / TEER of untreated). The average TEER of three replicates is shown and error bars represent the standard error (SEM).

Transport study

Transport studies were performed in transwell systems on Caco-2 barriers cultured for 21 days. After replacing the medium with pre-warmed fresh HBSS buffer (0.5 ml and 1.5 ml in the apical and basal chamber, respectively), the Caco-2 monolayer was incubated at 37 °C for 60 min in an orbital shaker (Titramax 1000) at 100 RPM. The HBSS was then removed and the inserts were transferred to a new 12 well plate. 0.5 ml 100 μg/ml 50 and 150 nm SiO_2-NPs dispersed in HBSS supplemented with 0%,

10%, 55% and 80% FBS in HBSS were added to the apical side of transwells and 1.5 ml fresh HBSS (without serum) added to the basolateral side. At the beginning of the experiment (t = 0), 100 µl aliquots were sampled from the apical chambers in order to determine the initial fluorescence of the applied nanoparticle dispersions. Similarly, at t = 0 and every hour up to 6 h, 100 µl aliquots were sampled from the basal chambers and replaced with 100 µl fresh HBSS buffer (without serum). Fluorescence measurements were performed on a Varioskan plate reader with excitation and emission settings at 490/516 nm. Calibration curves of fluorescence intensity as a function of nanoparticle concentration in the different serum concentrations tested were obtained and plotted as previously described [4]. Linear regression was applied to calculate the transported mass of nanoparticles present in the basolateral chamber at each time point, in the calculation correcting for the removal of 100 µl of nanoparticle solution and replacement with buffer. The percentage of transported mass was calculated by normalising the mass in the basolateral chamber for the initially applied mass in the apical chamber.

Figures

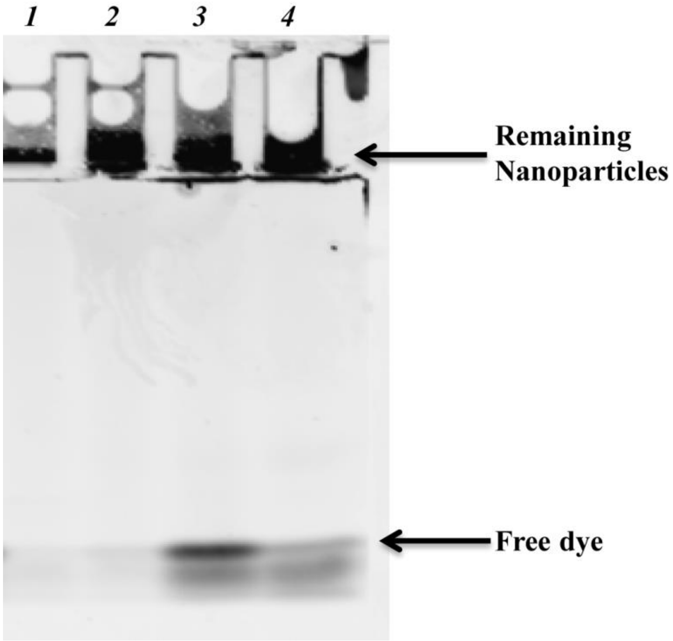

Figure S1: Fluorescence image of SDS-PAGE of green fluorescent silica nanoparticles used in this study. Lanes 1-2: freshly synthesized 50 and 150 nm SiO_2-NPs; Lanes 3-4: Commercially available 50 nm and 100 nm SiO_2-NPs. The same amount of particles (25 µg/ml) from the different batches was loaded on the gel. Eventual labile dye separates from the nanoparticles and migrates to the bottom of the gel due to its smaller size. In contrast, nanoparticles which are bigger than the gel pores remain in the loading wells. Using this method, the presence of eventual labile dye contaminating the particle stock can be detected. The synthesized 50 nm and 150 nm SiO_2-NPs showed very little dye release in comparison to what is observed for the commercial samples. The experiments in this study were therefore carried out using only batches synthesized in-house. As a further precaution, prior to experiments with cells, the nanoparticle stocks were cleaned of eventual labile dye by centrifugation and resuspension in fresh buffer, as described in the Experimental section.

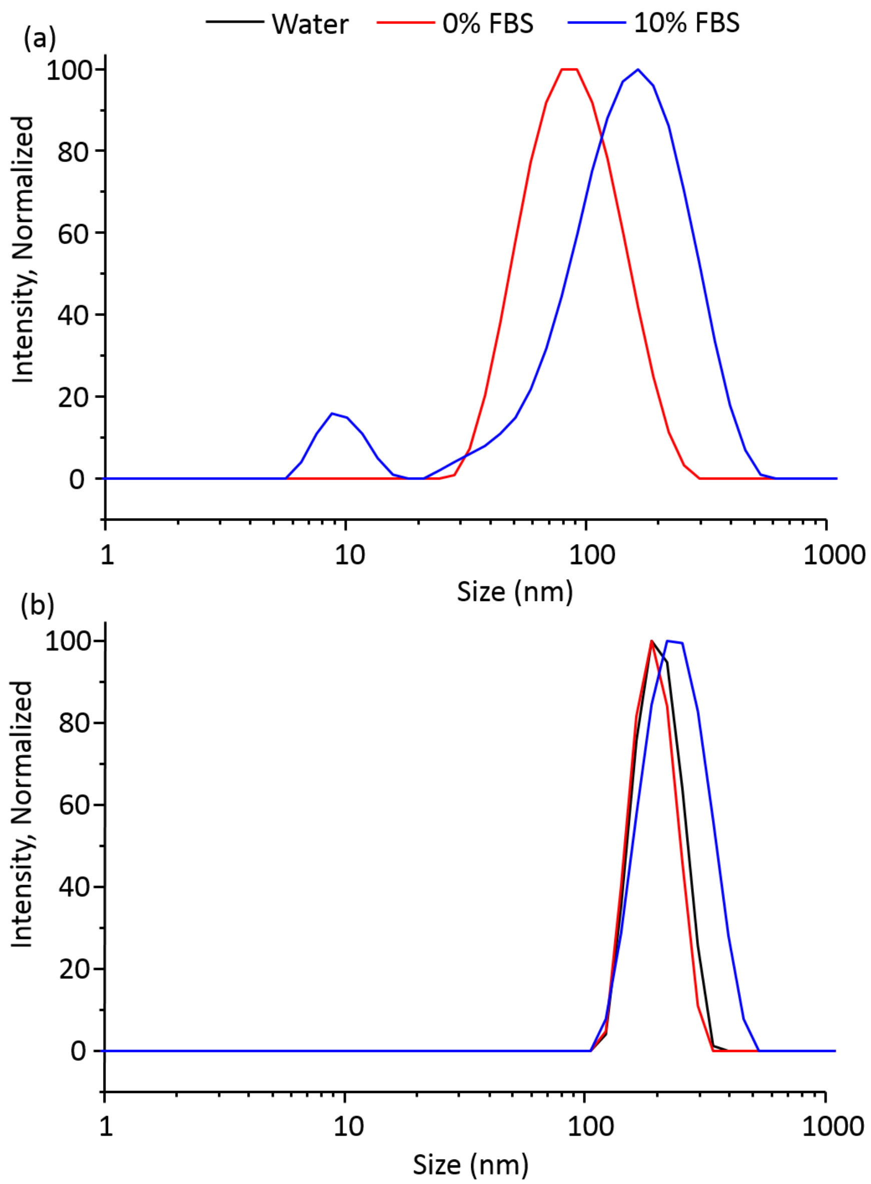

Figure S2: Physicochemical characterisation of the 50 nm and 150 nm SiO_2-NPs in different media using dynamic light scattering. Nanoparticle dispersions (100 µg/ml) in serum free HBSS and HBSS supplemented with 10% foetal bovine serum (FBS) were characterised by dynamic light scattering (DLS) as described in the Experimental section. The 150 nm were also measured in water. (a) 50 nm and (b) 150 nm SiO_2-NPs. The results are shown as the distributions by intensity, with corresponding averages reported in Table 1.

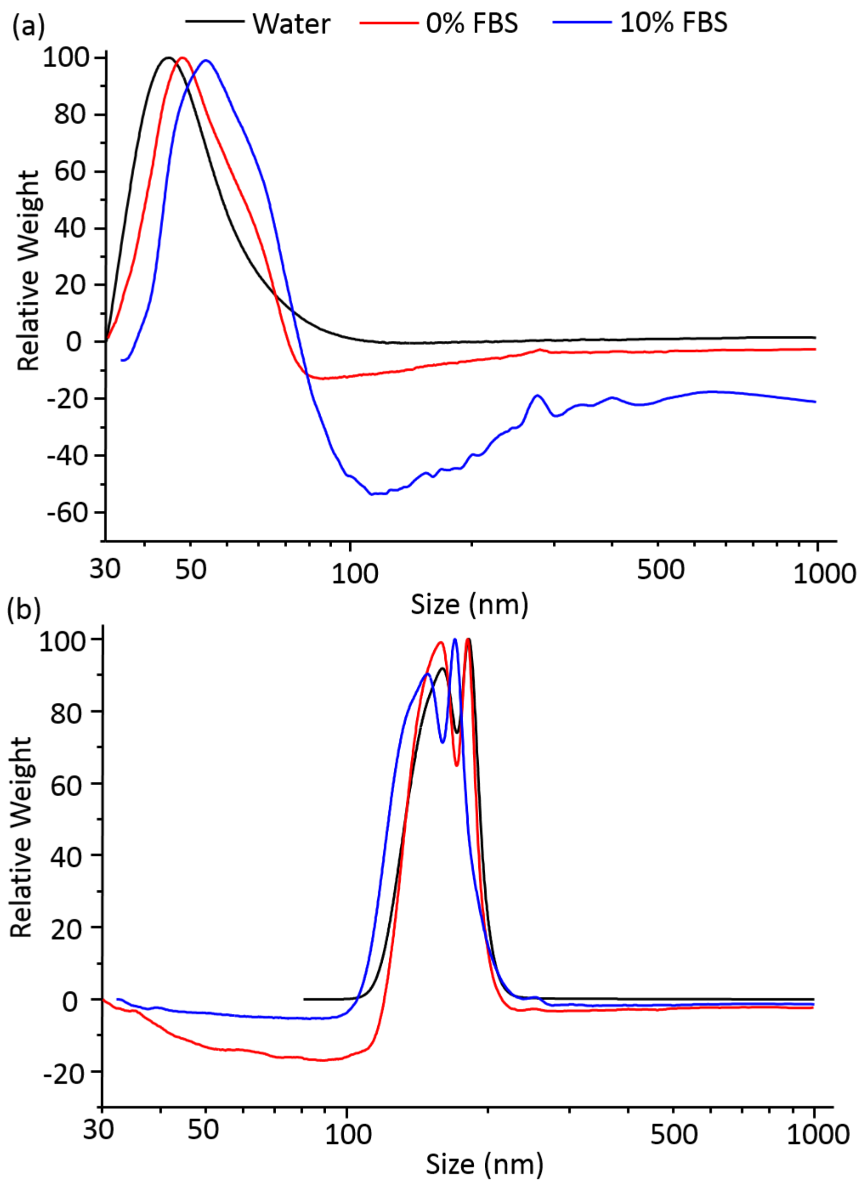

Figure S3: Physicochemical characterisation of the 50 nm and 150 nm SiO_2-NPs in different media using differential centrifugal sedimentation. Nanoparticle dispersions (100 µg/ml) in water, serum free HBSS and HBSS supplemented with 10% foetal bovine serum (FBS) were characterised by differential centrifugal sedimentation (DCS) as described in the Experimental section. The results show the distributions, with the corresponding peak positions reported in Table 1.

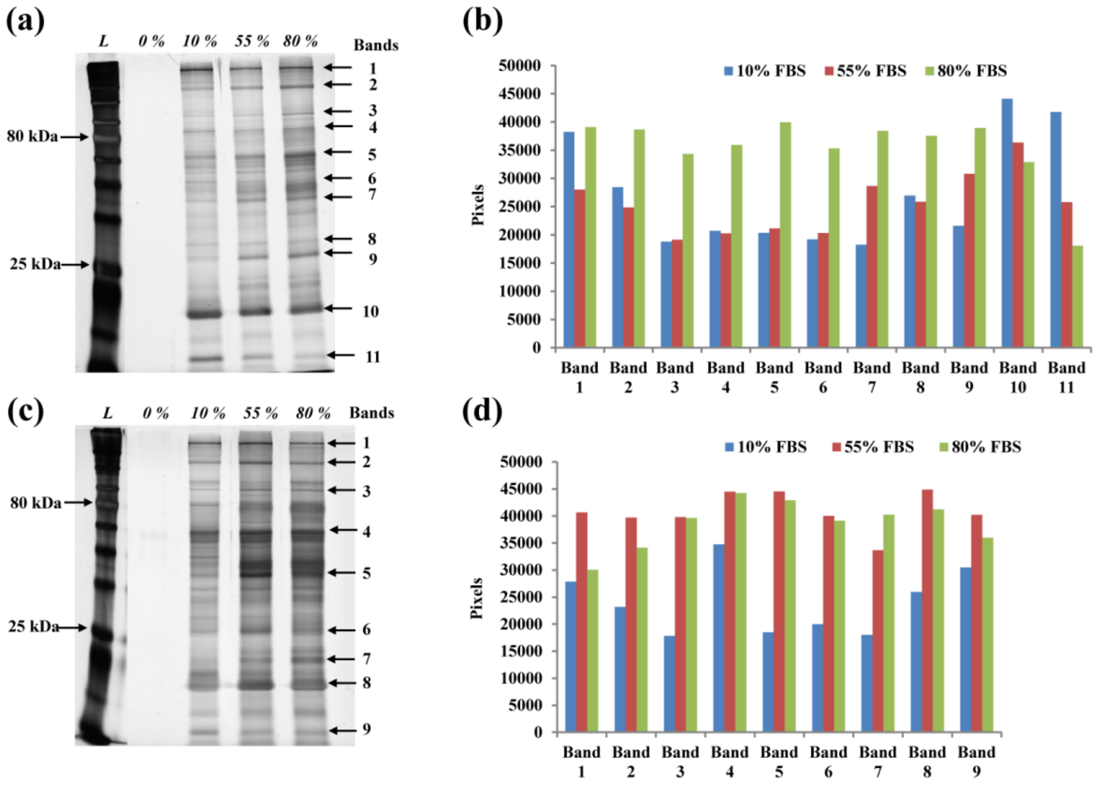

Figure S4: Qualitative and quantitative analysis of the protein corona formed on 50 nm and 150 nm SiO_2-NPs. The serum proteins adsorbed to 100 µg/ml (a) 50 nm and (c) 150 nm SiO_2-NPs (6 h incubation) were separated by SDS-PAGE and detected with silver staining, as described in the Supplementary Methods. From these gels, the intensity of typical protein bands (indicated by arrows in panels a and c) were analysed for both (b) 50 nm and (d) 150 nm SiO_2-NPs. In general, the abundance of the analysed corona proteins underwent quantitative changes as a function of medium serum concentration. For both particles, the adsorbed amount of several proteins increased monotonically (within implied detection limits) with overall serum concentration (for example, band 3-9 for 50 nm and band 3-7 for 150 nm SiO_2-NPs). These examples follow the naive assumption that incubation with more proteins results in more enriched protein coronas formed on the surface of the nanoparticles. Interestingly, more complicated behaviour can, however, be seen for other proteins. Thus, in some cases, adsorption decreases at the highest serum concentration (for

example, band 1-2 for 150 nm SiO_2-NPs), while other proteins exhibit the complete reverse behaviour and are less abundant as serum concentration increases (for example, band 10-11 for 50 nm SiO_2-NPs). This is consistent with previous reports for similar nanoparticles in plasma [5], where it was also found that the corona composition varied with protein content. Such observations demonstrate the complexity of the corona formation, while emphasizing the importance of fully specifying the exposure conditions in nanoparticle studies, since exposure to different coronas may result in different outcomes on cells.

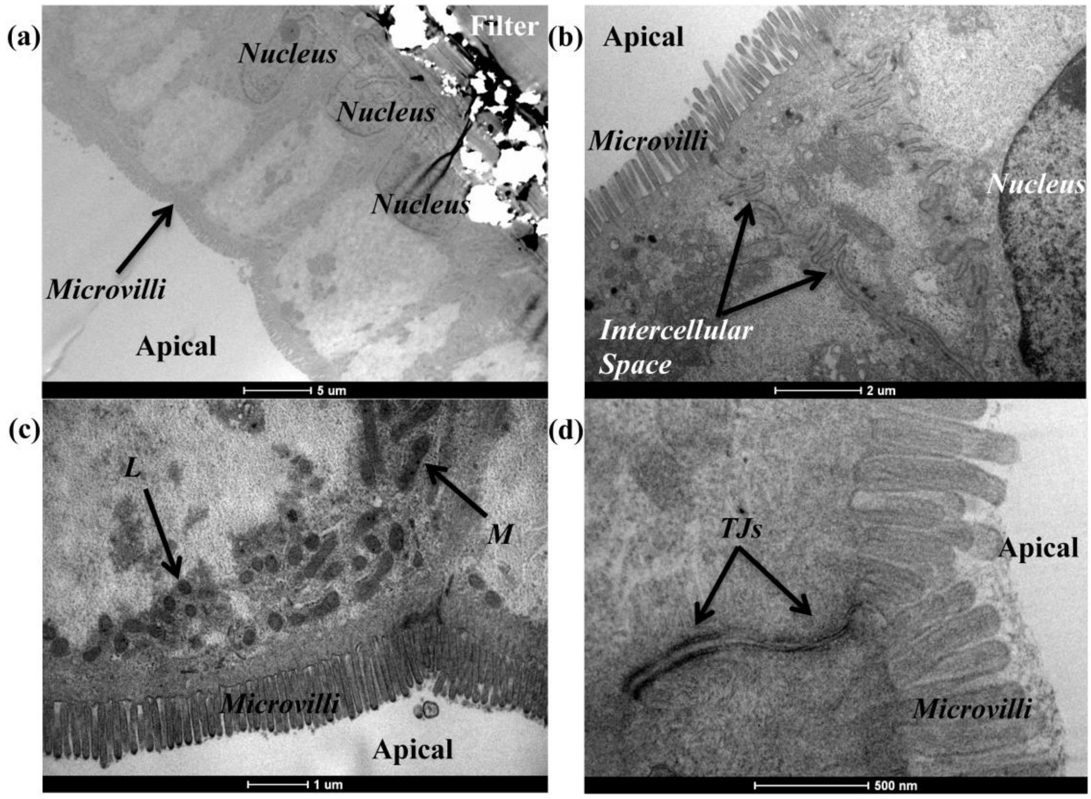

Figure S5: Details of Caco-2 barrier morphology and organisation. TEM images of Caco-2 barriers cultured for 21 days as described in the Experimental section. The results confirm that a well polarised cell monolayer with apical microvilli and nuclei in basal position was obtained, and that tight junctions were expressed between the confluent cells. Arrows indicate some of the internal structures. Abbreviations: *L*, lysosome; *M*, mitochondrion; *TJs*, tight junctions.

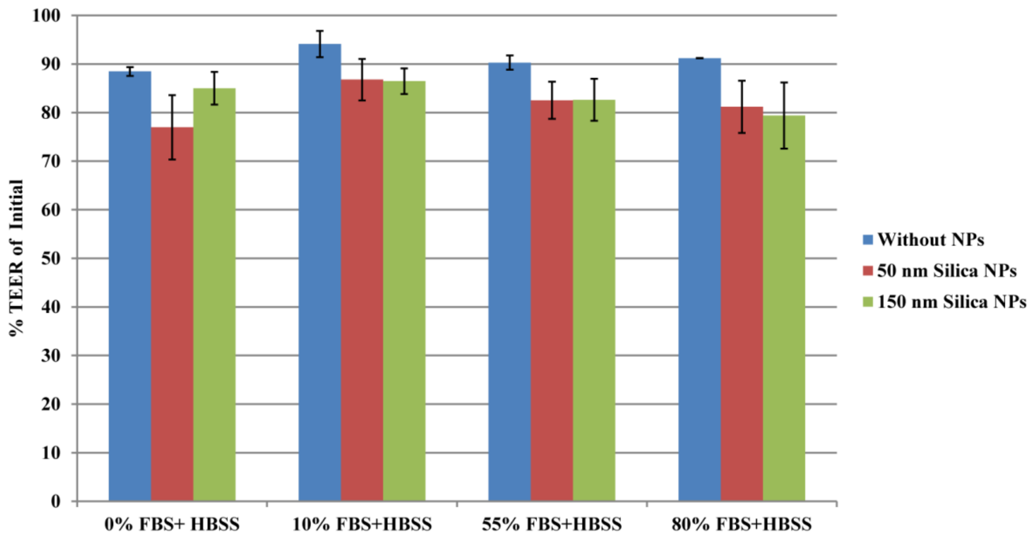

Figure S6: Characterisation of Caco-2 barrier integrity after exposure to SiO_2-NPs. Change in transepithelial electrical resistance (TEER) of Caco-2 barriers (cultured for 21 days) following 6 h exposure to nanoparticle-free media and media containing 100 µg/ml 50 nm or 150 nm SiO_2-NPs as a function of serum concentration. The change in TEER is presented relative to the initial (prior to exposure) value. Error bars represent the standard error of the mean of 3 replicates from four independent experiments. Note that there are necessarily charged objects present (the nanoparticles and proteins) in the experiments.

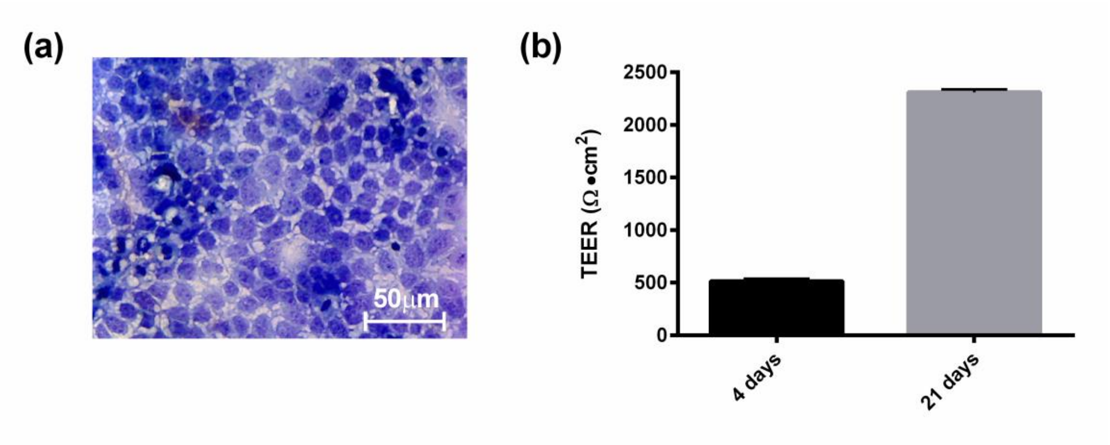

Figure S7: Development between 4 and 21 days of culturing Caco-2 barriers. (a) Stained with toluidine blue, Caco-2 barriers cultured for 4 days already exhibit a confluent barrier-like appearance under observation by light microscopy (10X magnification). (b) Nevertheless, signficant TEER differences may be seen between 4 and 21 days of culturing Caco-2 cells on 6-well transwell membranes (Corning, growth area 4.67cm^2).

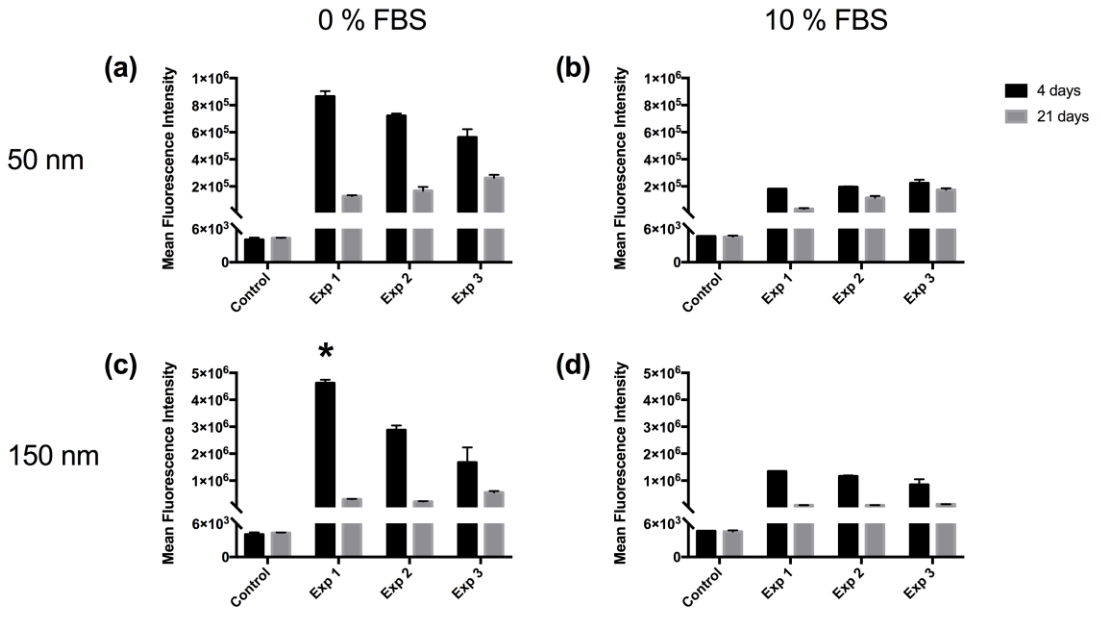

Figure S8: 50 nm and 150 nm SiO_2-NP association with Caco-2 barriers. Caco-2 barriers cultured for 4 and 21 days were exposed for 6 h to (a-b) 50 nm and (c-d) 150 nm SiO_2-NPs (100 µg/ml) in the absence (a and c) and presence of 10% serum (b and d), prior to cell fluorescence measurements by flow cytometry. Results are presented as the mean cell fluorescence intensity (due to nanoparticles) with error bars representing the standard error of the mean of 3 technical replicates. The different results are from three independent experiments, exhibiting quantitative differences but, generally, the same trend of a larger nanoparticle association to barriers cultured for 4 days compared to those cultured for 21 days, as well as a trend of larger association in the absence of serum. The asterisk (*) in panel c indicates that the actual value is higher than the one measured and presented due to the limited range of the instrument. Note that the two nanoparticles are loaded with different amounts of fluorescent dye so no (direct) absolute comparison may be made between the two sizes.

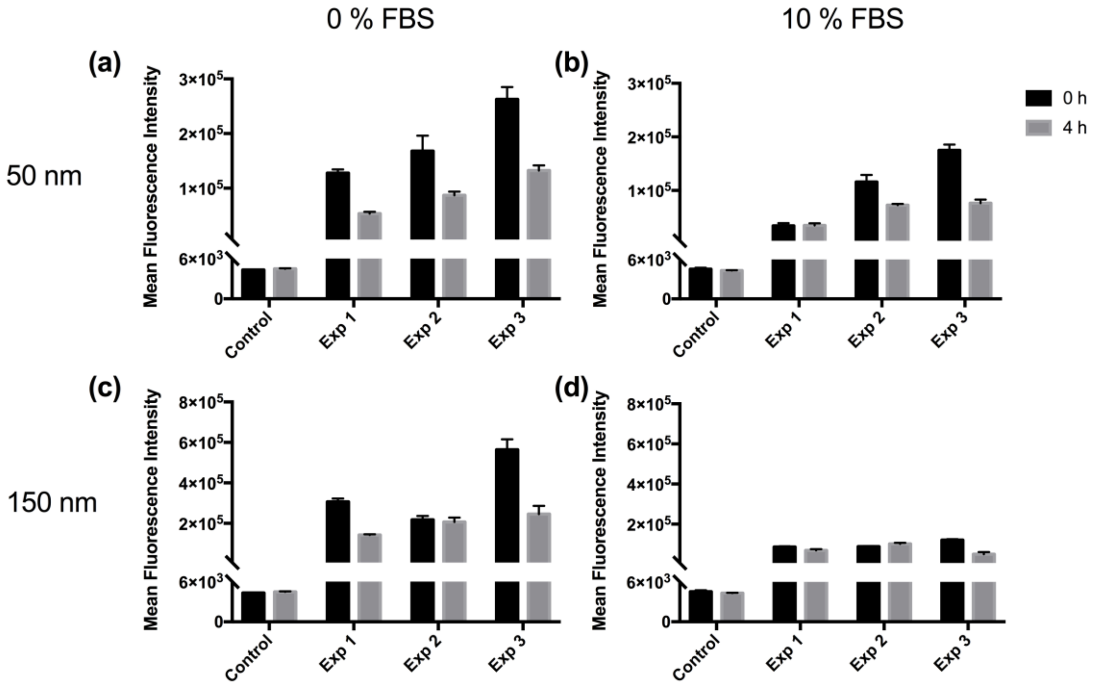

Figure S9: Nanoparticle association with Caco-2 barriers after removal of nanoparticle source. Caco-2 barriers (cultured for 21 days) were exposed for 6 h to (a-b) 50 nm and (c-d) 150 nm SiO_2-NPs (100 µg/ml) in the absence of serum (a and c) and the presence of 10% FBS (b and d), after which the nanoparticle source was removed and the cells further cultured in nanoparticle-free medium for 4 h prior to cell fluorescence measurements by flow cytometry. Results are presented as the mean cell fluorescence intensity (due to nanoparticles) with error bars representing the standard error of the mean of 3 technical replicates. The different results are from three independent experiments, exhibiting quantitative differences but, generally, the same trend of a lower nanoparticle association after culturing in the absence of nanoparticles.

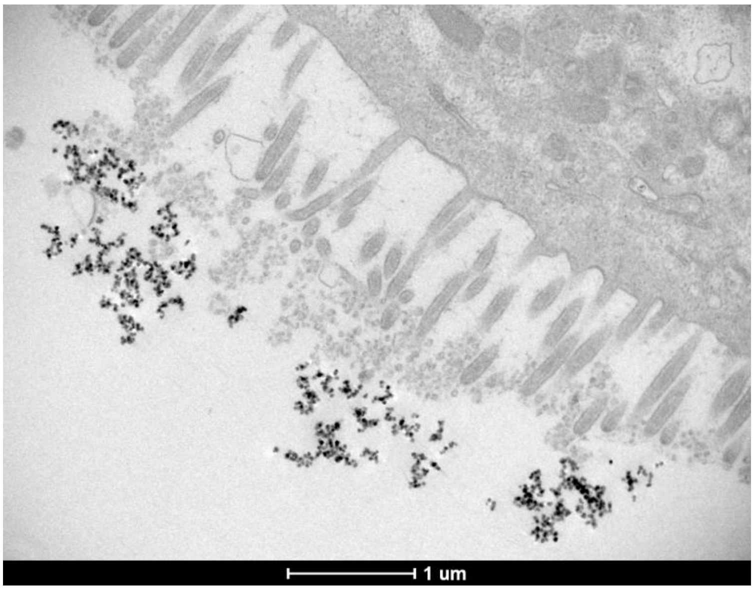

Figure S10: Surface association of 50 nm SiO_2-NPs exposed for 6 h to Caco-2 barriers cultured for 21 days. A large amount of nanoparticles was found outside the cells, while very few were found intracellularly.

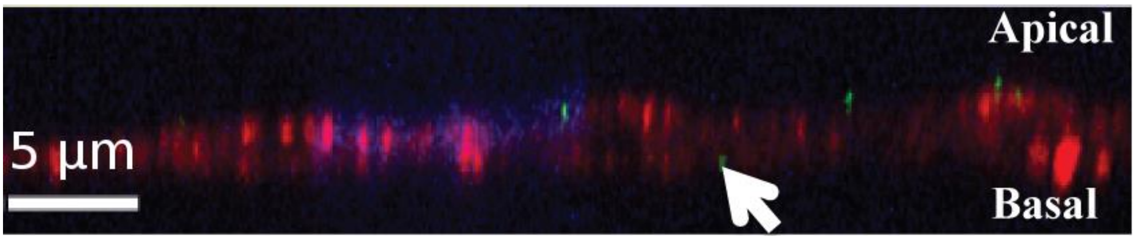

Figure S11: Confocal fluorescence image of a cross-section of Caco-2 barriers exposed to 50 nm SiO_2-NPs. Caco-2 barriers were exposed to 100 µg/ml 50 nm SiO_2-NPs for 6 h in medium with 80 % FBS. The nanoparticles are shown in green. Lysosomes and nuclei were stained by LAMP1 antibody (red) and DAPI (blue), respectively. The arrow indicates nanoparticle fluorescence close to the basal membrane, which could be suggestive of a nanoparticle (or several within one fluorescent spot) potentially having or being about to translocate the barrier.

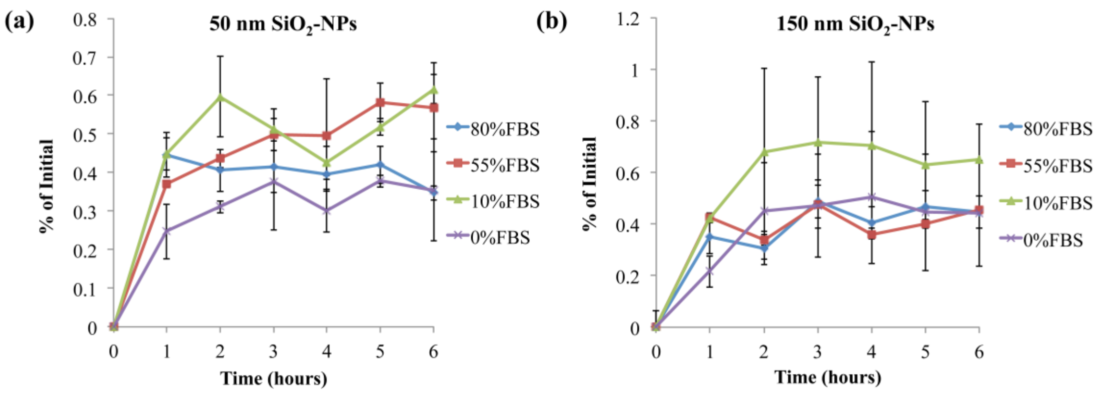

Figure S12: Nanoparticle transport across Caco-2 barriers cultured for 21 days. Transport of (a) 50 nm and (b) 150 nm SiO_2-NPs at different serum concentration across Caco-2 barriers as a function of time. The results are presented as the proportion of mass transported across the barrier relative to the initial dose on the apical side. Error bars represent the standard error over 3 independent replicates.

References

1. Shapero, K.; Fenaroli, F.; Lynch, I.; Cottell, D. C.; Salvati, A.; Dawson, K. A. *Mol Biosyst* **2011,** *7,* 371-378.

2. Santos, T.; Varela, J.; Lynch, I.; Salvati, A.; Dawson, K. A. *Small* **2011,** *7,* 3341-3349.

3. Salvati, A.; Åberg, C.; dos Santos, T.; Varela, J.; Pinto, P.; Lynch, I.; Dawson, K. A. *Nanomedicine* **2011,** *7,* 818-826.

4. Nic Raghnaill, M.; Brown, M.; Ye, D.; Bramini, M.; Callanan, S.; Lynch, I.; Dawson, K. A. *Eur J Pharm Biopharm* **2011,** *77,* 360-367.

5. Monopoli, M. P.; Walczyk, D.; Campbell, A.; Elia, G.; Lynch, I.; Baldelli Bombelli, F.; Dawson, K. A. *Journal of the American Chemical Society* **2011,** *133,* 2525-2534.
